# *KLRC3*, a Natural Killer receptor gene, is a key factor involved in glioblastoma tumourigenesis and aggressiveness

**DOI:** 10.1111/jcmm.12960

**Published:** 2016-09-19

**Authors:** Mathilde Cheray, Barbara Bessette, Aurélie Lacroix, Carole Mélin, Soha Jawhari, Sandra Pinet, Elise Deluche, Pierre Clavère, Karine Durand, Ricardo Sanchez‐Prieto, Marie‐Odile Jauberteau, Serge Battu, Fabrice Lalloué

**Affiliations:** ^1^ EA3842 Homéostasie Cellulaire et PathologiesFaculty of Medicine of LimogesUniversity of LimogesLimogesFrance; ^2^ Oncology DepartmentUniversity HospitalLimogesFrance; ^3^ Immunology Lab.University HospitalLimogesFrance; ^4^ Radiotherapy DepartmentUniversity HospitalLimogesFrance; ^5^ Pathology and AnatomyCBRSLimogesFrance; ^6^ Laboratorio de Oncología MolecularCentro Regional de Investigaciones BiomédicasUniversidad de Castilla‐La Mancha/PCyTA/Unidad de Biomédicina UCLM‐CSICAlbaceteSpain; ^7^ Present address: Department of Oncology‐PathologyCancer Centrum Karolinska (CCK)R8:03, Karolinska InstitutetSE‐171 76StockholmSweden

**Keywords:** glioblastoma, biomarker, NKG2E/KLRC3, tumourigenicity

## Abstract

Glioblastoma is the most lethal brain tumour with a poor prognosis. Cancer stem cells (CSC) were proposed to be the most aggressive cells allowing brain tumour recurrence and aggressiveness. Current challenge is to determine CSC signature to characterize these cells and to develop new therapeutics. In a previous work, we achieved a screening of glycosylation‐related genes to characterize specific genes involved in CSC maintenance. Three genes named *CHI3L1*,*KLRC3* and *PRUNE2* were found overexpressed in glioblastoma undifferentiated cells (related to CSC) compared to the differentiated ones. The comparison of their roles suggest that *KLRC3* gene coding for NKG2E, a protein initially identified in NK cells, is more important than both two other genes in glioblastomas aggressiveness. Indeed, *KLRC3* silencing decreased self‐renewal capacity, invasion, proliferation, radioresistance and tumourigenicity of U87‐MG glioblastoma cell line. For the first time we report that *KLRC3* gene expression is linked to glioblastoma aggressiveness and could be a new potential therapeutic target to attenuate glioblastoma.

## Introduction

Glioblastoma multiforme (GBM) is the most common form of gliomas and the most aggressive brain tumour. Patients with GBMs have a poor prognosis and median survival is around 15 months because of tumour recurrence in spite of surgery, radiotherapy and concomitant chemotherapy 1. Glioblastoma multiformes are highly heterogeneous tumours where the most resistant and aggressive cells required for tumour recurrence are the most undifferentiated cells considered as cancer stem cells (CSC) 2, 3. Cancer stem cells characterization still remains difficult because of the absence of specific marker and their restricted cell population.

Previously, we demonstrated a correlation between specific glycosylation‐related genes overexpression and undifferentiated glioblastoma cells related to CSC 4. In this work we observed an overexpression of three specific glycosylation‐related genes, *CHI3L1*,* KLRC3* and *PRUNE2* in CSC from cancer cell lines and human primary tumour cells. *PRUNE2* was recently known to be expressed in melanoma and prostate cancer in which it is associated with AP2 protein involved in vesicle trafficking 5. CHI3L1 is already described in glioblastoma model where an anti‐CHI3L1 is proposed as new adjuvant therapy in clinic 6. Unlike PRUNE2 and CHI3L1 proteins, *KLRC3* gene coding for NKG2E protein, has never been reported in cancer. Indeed, this NK cell receptor is only described as an essential protein involved in viral resistance 7. The putative role of *KLRC3* is still unknown in glioblastoma aggressiveness. In the present study, the effect of *KLRC3* extinction is compared to those of the two other genes *CHI3L1* and *PRUNE2* in a human glioblastoma cell line. Although silencing of *CHI3L1*,* KLRC3* and *PRUNE2* decrease cell proliferation, migration, clonogenicity and tumourigenesis, our results demonstrate that *KLRC3* is of prime importance for glioblastoma cells aggressiveness and their ability to promote cancer progression. These findings suggest that NKG2E is a potential new target for the development of new therapy against glioblastoma.

## Materials and methods

### Cell culture conditions

U87‐MG human glioblastoma cell line was obtained from American Type Culture Collection (ATCC/LGC promochem, Molsheim, France). The cells were maintained in MEM with Earl's salts (Gibco BRL, Life Technologies, Paisley, UK) supplemented with 10% foetal calf serum, 1.5 g/l sodium bicarbonate, 1% non‐essential amino acids, 2 mM sodium pyruvate, 50 units/mL penicillin, 50 units/mL streptomycin and 2 mM L‐Glutamine. Cells were grown in 75 cm^2^ flasks (Nunc Fisher Bioblock Scientific, Illkirch, France) at 37°C in a humidified 5% CO_2_–95% air incubator.

### Gene silencing by lentiviral transfection of shRNA

ShRNA for *CHI3L1*,* KLRC3* and *PRUNE2* in pLKO‐PURO vectors were purchased from Sigma‐Aldrich (Saint‐Quentin‐Fallavier, France). Two to three clones were tested for each shRNA (except for shPRUNE2 which was already validated) (Fig. S1). Cell lines were established by lentiviral infection using the packaging cell line HEK‐293T. HEK‐293T cells were transfected with lipofectamine with MISSION^®^ Lentiviral Packaging Mix and pLKO‐*CHI3L1*, pLKO‐*KLRC3*, pLKO‐*PRUNE2* or empty pLKO plasmids (leading to U87‐MG KO cells named shCHI3L1, shKLRC3, shPRUNE2 and pLKO respectively) (Invitrogen, Life Technologies, Illkirch, France). Supernatant was removed after 48 hrs and used for infecting U87‐MG cells for 6 hrs in the presence of polybrene (Sigma‐Aldrich). After 2 days, cells were selected using medium with 1 μg/ml of Puromycin (Cayla – InvivoGen, Toulouse, France) and shRNA efficiency was controlled by western blotting analysis.

### Western Blotting and semi‐quantification

Total lysate from different cell lines were obtained using lysis buffer [50 mM Tris‐Cl (pH 8.0), 150 mM NaCl, 1% NP‐40, 0.5% sodium deoxycholate, 0.1% SDS, 100 μg/ml phenylmethylsulphonyl fluoride, 0.5 μg/ml leupeptin, and 1 μg/ml aprotinin]. For each sample, 40 μg of proteins were loaded and separated onto gradient SDS‐polyacrylamide gel (Bio‐Rad, Marnes‐La‐Coquette, France) for 1 hr 30 at 120 V, then transferred on PVDF membrane (Bio‐Rad). After a blocking step (nonfat milk), membranes were incubated overnight at 4°C with primary antibodies diluted in blocking solution (anti‐CHI3L1 from Abcam, Paris, France; anti‐PRUNE2 and anti KLRC3 from Abnova, Paris, France, anti‐DAP12 from Cell Signaling, Paris, France), followed by incubation with the appropriate secondary HRP‐conjugated antibodies. Blots were developed with Immobilon Western chemiluminescent HRP substrate (Millipore Fontenay‐Sous‐Bois, France) and analysed with G‐Box (Ozyme; Fisher Scientific, Illkirch, France). Band intensities were determined by densitometry using ImageJ software (NIH, Bethesda, MD, USA).

### Clonogenic assay

Clonal agar culture was performed in a double‐layer agar system: 1 ml of basal layer culture with a final 0.5% agar concentration was prepared in 6‐wells plates. Then, 2.5 × 10^3^ U87‐MG, shCHI3L1, shKLRC3 or shPRUNE2 viable cells (cultivated prior in defined medium as previously described in Ref. 13) in 1 ml of upper layer medium (0.35% agarose) were overlaid on the preformed basal layer. 1 ml of medium was added on the top of the agar‐agarose layers and changed for fresh medium every 3 days. Plates were incubated for 30 days at 37°C in a humidified 5% CO_2_ environment. Only colonies visible to the naked eye were counted. Each cell line was analysed in triplicates.

### Cell radiation

U87‐MG cells and shRNA cells were seeded in 96 well plate at a density of 10,000 cells per well in 200 μl normal medium. Cells were irradiated with a total dose of 7 Gy using a ^192^Ir projector after 24 hrs of culture. Then, 72 hrs after radiation, apoptosis was evaluated by quantification of cytoplasmic soluble nucleosomes (Cell Death Detection ELISA kit; Roche, Meylan, France) and proliferation was quantified by BrdU incorporation (Cell Proliferation Assay kit; Cell Signaling Technology, Danvers, MA, USA) according to the manufacturer's instructions.

### Cell invasion assay

Cell invasion ability was assessed using BD BioCoat Matrigel Invasion Chambers according to the manufacturer's instructions. Invading cells were stained with calcein and fluorescence was measured using a fluorescent microplate reader at 494/517 nm (Twinckle LB970; Berthold, Thoiry, France). Images were taken under a fluorescent binocular (Leica Microsystems, Nanterre, France).

### Tumour xenografts in mice

Female nude mice (Charles River, Ecully, France) were subcutaneously injected with 3 × 10^6^ pLKO, shCHI3L1, shKLRC3 or shPRUNE2 cells in 0.2 ml of PBS. Ten mice per group were evaluated twice a week to measure tumour volumes (V = 0.5 (length × width^2^)) during 5 weeks before the the animals were killed (*n* = 40). The experimental procedures using animals were in accordance with the guidelines of Institutional Animal Care and the French National Committee of Ethics.

### Primary cell culture

All patients‐derived tissues (tissue sections and primary cell culture) were obtained from Tumor Biobank of Limoges University Hospital according to Ethics Committee of the Limoges University Hospital (Protocol 141‐2014‐08). Cell dissociation was performed mechanically. Cells were cultured in NSA‐H medium (StemCell, Grenoble, France) with 10 ng/ml βFGF, 20 ng/ml EGF, and 1 mg/ml Heparin, until appearance of floating gliomaspheres. Half of the culture medium was renewed each week. Once the cultures stabilized, the gliomaspheres were routinely dissociated once a week and seeded in fresh medium.

### Immunocytochemistry

Smears of gliomaspheres were performed on glass slide. Cells were fixed for 10 min. using 4% paraformaldehyde at room temperature. Cell smears were washed in PBS. Immunocytochemistry stainings were performed using anti‐DAP12 (clone D7G1X; Cell Signaling) or anti‐KLRC3 (Abnova) antibodies in a Leicabondmax (LeicaBiosystem, Nanterre, France, financed by SATT Grand Centre) according to protocols supplied by the manufacturers.

### Immunhistochemistry

After representative areas of tumour tissue were selected using hematoxylin eosin saffron‐stained sections, 5 μm‐thick sections were cut from paraffin‐embedded blocks. Sections were incubated with Ki67 antibody (clone MiB‐1, 1/200; DakoCytomation, Glostrup, Denmark) for mice tissue sections or DAP12 antibody (clone D7G1X; Cell Signaling) for human tumour sections. Sample slides were processed automatically (BenchMark XT ICH/ISH; Ventana Medical Systems, OroValley, USA) according to protocols supplied by the antibody manufacturers.

### Proteome array

The Proteome Profiler Human Phospho‐MAPK Array Kit (R&D Systems, Minneapolis, USA) was used to screen the phosphorylation status of 26 kinases involved in cell proliferation. Procedure was performed according to the manufacturer's instructions. Briefly, protein lysate from pLKO, shKLRC3 and GBM1 were obtained using the lysis buffer provided in the kit. Protein lysates were incubated with detection antibody cocktail on membranes after saturation. Revelation was performed using chemi reagent mix. Samples were analysed with G‐Box (Ozyme; Fisher Scientific). Dots intensities were determined by densitometry using ImageJ software (NIH).

### Statistical analysis

All the comparisons between groups were performed by a one‐way anova with Statview 5.0 software (Abacus Concepts, Piscataway, USA). Differences were considered significant at a *P* < 0.05.

## Results

### KLRC3 silencing reduces proliferation and enhances apoptosis and radiosensitivity of glioblastoma cells *in vitro*

**Figure 1:**
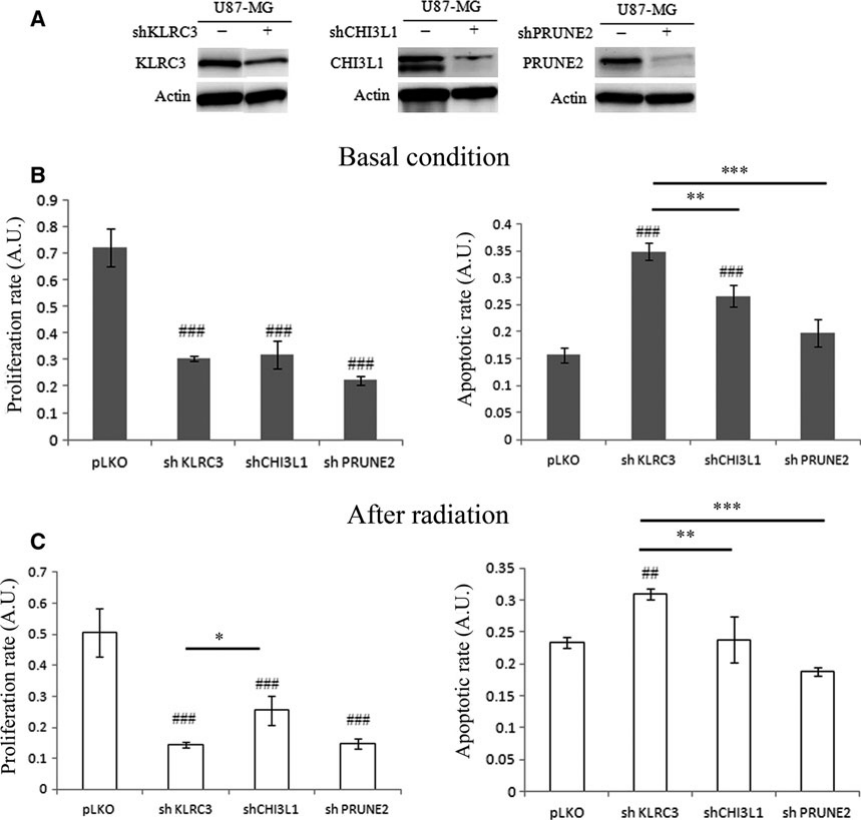
*KLRC3* silencing decreases U87‐MG cell proliferation rate and enhances apoptosis before and after radiation. (**A**) KLRC3, CHI3L1 and PRUNE2 protein levels studied by Western Blot analysis in U87‐MG control cells (‐) and U87‐MG shRNA cells (+). Actin is used as loading control. (**B**) Left panel: Cell proliferation rate of control (pLKO) and shRNA cells mean ± S.E.M. determined by BrdU incorporation in basal condition. ^###^ *P* < 0.001 pLKO *versus* shKLRC3, shCHI3L1, shPRUNE2. Right panel: Apoptotic cell death of control (pLKO) and shRNA cells mean ± S.E.M. determined by ELISA method in basal condition.^ ###^ *P* < 0.001 pLKO *versus* shKLRC3, shCHI3L1; **P < 0.01 shKLRC3 *versus* shCHI3L1; ***P < 0.001 shKLRC3 *versus* shPRUNE2. (**C**) Left panel: Cell proliferation rate of control (pLKO) and shRNA cells mean ± S.E.M. determined by BrdU incorporation after cell radiation. ^###^ *P* < 0.001 pLKO *versus* shKLRC3, shCHI3L1, shPRUNE2; *P < 0.05 shKLRC3 *versus* shCHI3L1. Right panel: Apoptotic cell death of control (pLKO) and shRNA cells mean ± S.E.M. determined by ELISA method after cell radiation. ^##^ *P* < 0.01 pLKO *versus* shKLRC3; **P < 0.01 shKLRC3 *versus* shCHI3L1; ***P < 0.001 shKLRC3 *versus* shPRUNE2. A.U.: Arbitrary Units.

Based on our previous study describing an overexpression of three glycosylation‐related genes, *KLRC3*,* CHI3L1* and *PRUNE2* in glioblastomas undifferentiated cells related to CSC [4], we assessed whether these genes are closely linked to glioma tumourigenesis mechanisms. *KLRC3*,* CHI3L1* and *PRUNE2* gene extinction was performed by shRNA. The knockdown efficiency was evaluated by western blot (Fig. 1A) and reached only 52% for KLRC3, whereas it reached 76% and 79% for CHI3L1 and PRUNE2, respectively. The effect of these three gene extinctions on cell proliferation ratio was evaluated by BrdU incorporation. A significant cell proliferation decrease is observed in the three shRNA cell lines compared to the control one (pLKO) (Fig. 1B, left panel). Moreover, this decrease is more important in shKLRC3 cells (*P* < 0.001) and shPRUNE2 cells (*P* < 0.001) (Fig. 1B, left panel), suggesting that these two genes might be involved in U87‐MG cell proliferation. Although the inactivation of *KLRC3* was obtained at a lesser extent that those of the two other genes (Fig. 1A), cell proliferation was drastically reduced in shKLRC3 cells (Fig. 1B). We then analysed the effect of these three genes extinction on U87‐MG apoptosis ratio in normal conditions. Among the three shRNA, only *KLRC3* and *CHI3L1* extinctions lead to a significant increase in U87‐MG cells basal apoptosis compared to pLKO control cells (Fig. 1B right panel). Moreover, the cell death ratio is significantly increased after *KLRC3* extinction compared to shCHI3L1 or shPRUNE2 (Fig. 1B right panel).

Radiotherapy, the main glioma treatment is often impaired because of tumour cells radioresistance leading to recurrence. In this context, we suggested that *KLRC3* could be involved in glioblastoma cells radioresistance. Therefore, the proliferation rate of the shRNA cells was analysed after *in vitro* exposure to 7 Gy of total radiation in comparison to pLKO cells as control (Fig. 1C, left panel). As expected, we observed a significant decrease in cell proliferation in the three shRNA cell lines compared to the pLKO control cells (Fig. 1C, left panel). More interestingly, the *KLRC3* extinction leads to a significant decrease in cell proliferation compared to shCHI3L1 cells suggesting that shKLRC3 cells are more sensitive to radiation than shCHI3L1 cells. To validate this hypothesis, we analysed the apoptosis induction after cell radiation in our three shRNA cell lines. Cell radiation induces a significant increase in the apoptosis ratio only in shKLRC3 cells compared to control cells (*P* < 0.01) (Fig. 1C, right panel). This result demonstrates that among the three analysed genes, only *KLRC3* is involved in U87‐MG cell sensitivity to radiation treatment. This is also confirmed by the fact that the only shRNA cell line that showed a significant decrease in cell proliferation after radiation is the shKLRC3 cell line (compared to non‐irradiated shKLRC3 cells) (*P* < 0.01, data not shown).

### KLRC3 silencing decreases glioblastoma cell migration and clonogenicity

**Figure 2:**
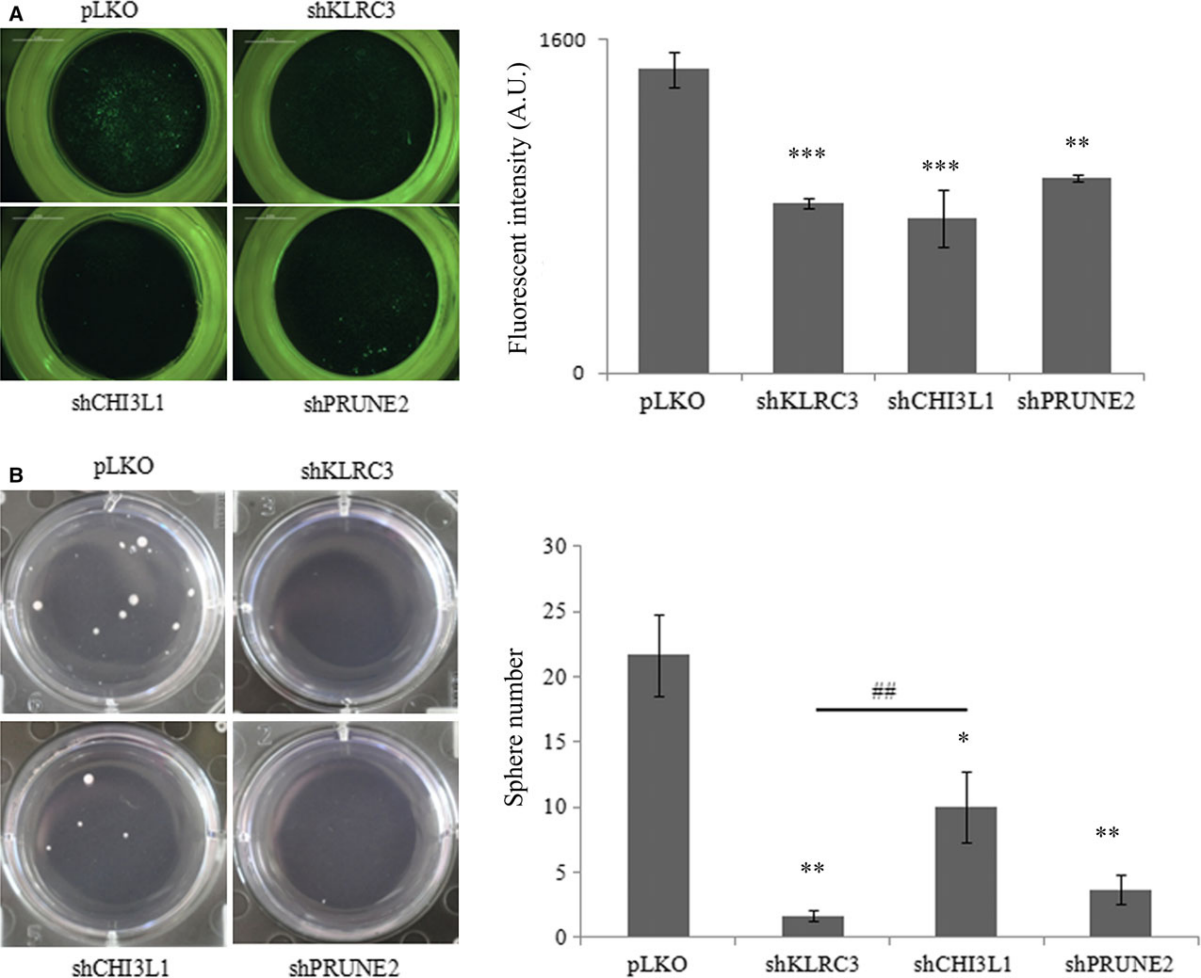
*KLRC3* silencing decreases U87‐MG migration ability and clonogenicity. (**A**) Cell invasion assessed using matrigel chambers. Left panel: photos of migrating cells through matrigel stained with calcein. Right panel: Fluorescence intensity mean ± S.E.M. shRNA cells *versus* pLKO: **P < 0.01; ***P < 0.001. A.U.: Arbitrary Units. (**B**) Cell clonogenicity was determined using double layer agar‐system. Left panel: pictures showing gliomasphere formation after 30 days of culture. Right panel: graph show gliomasphere number mean ± S.E.M. *P < 0.05 shCHI3L1 *versus* pLKO; **P < 0.01 shKLRC3 *versus* pLKOand shPRUNE2 *versus* pLKO; ^##^ *P* < 0.01 shKLRC3 *versus* shCHI3L1.

Glioblastomas cells have the ability to migrate to invade the healthy tissue in the brain. To analyse the role of *KLRC3*,* CHI3L1* and *PRUNE2* genes in the migration ability of glioblastoma cells we used a matrigel invasion chamber system. The three shRNA cell lines (shCHI3L1, shKLRC3 and shPRUNE2) exhibited a significant decrease in their invasion ability, compared to the control cells (Fig. 2A).

Then, to assess the impact of the three different gene extinction on glioblastoma self‐renewal properties, the ability of the different cells to form gliomaspheres has been studied in a double‐layer agar system. The number of colonies was significantly reduced for the shRNA cells compared to control (shCHI3L1: *P* < 0.05; shPRUNE2: *P* < 0.01; shKLRC3: *P* < 0.01) (Fig. 2B). Moreover, the self‐renewal property of shKLRC3 cells was significantly lower than shCHI3L1 cells (*P* < 0.01), but no significant difference was observed in comparison with shPRUNE2 cells. Although the three genes seem to be involved in invasive mechanisms and self‐renewal ability, *KLRC3* and *PRUNE2* might play a key role in maintaining these properties (related to CSC properties) since their inactivation block significantly self‐renewal properties in comparison to shCHI3L1 cells.

### Tumour growth is inhibited by the inactivation of KLRC3 gene *in vivo*

**Figure 3:**
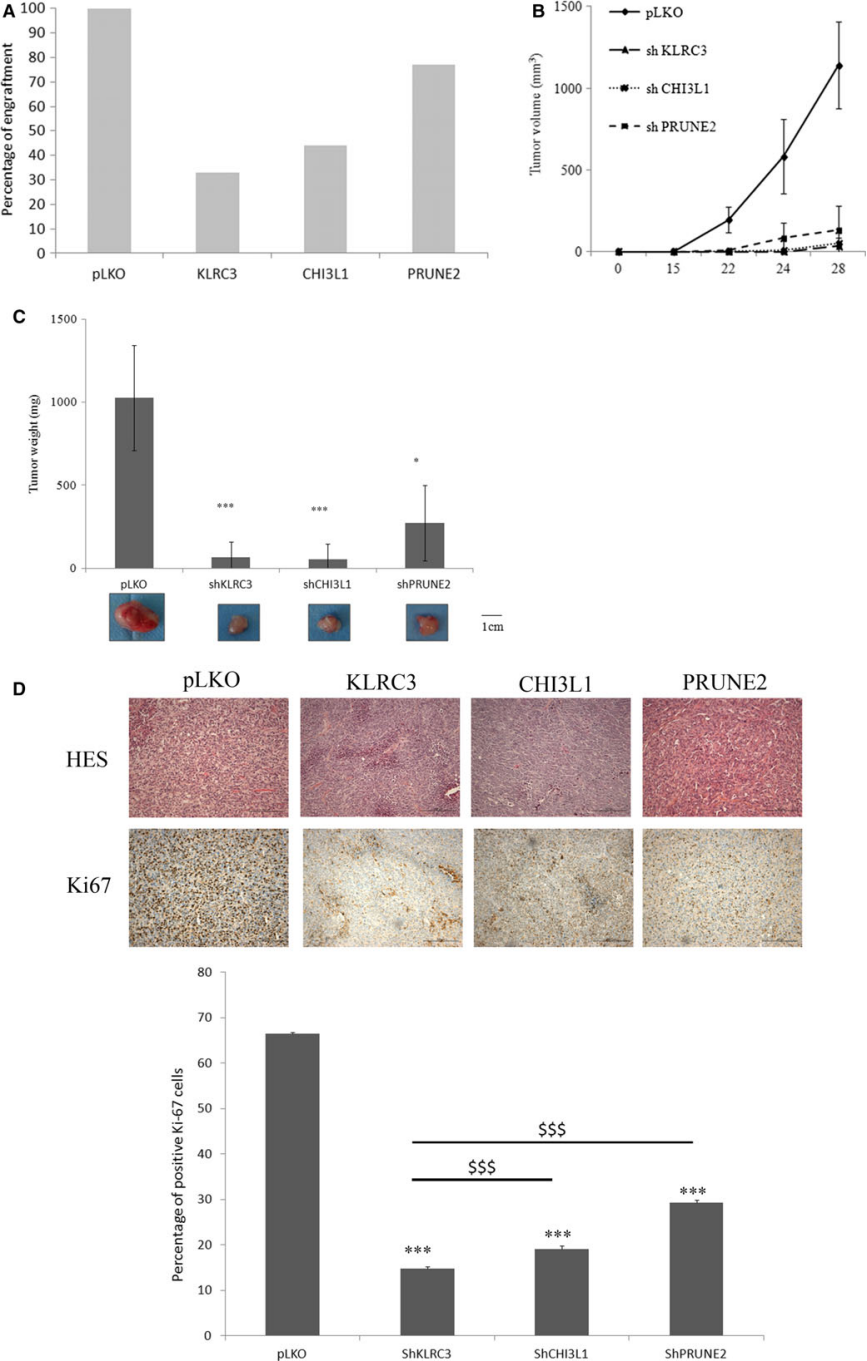
U87‐MG tumourigenesis is impaired in glioblastoma shRNA cells. (**A**) Percentage of formed tumour observed after killing of Nude mice engrafted with pLKO or shRNA cells. (**B**) Tumour volumes have been measured during 1 month after cell xenografts in Nude mice and graph represents means ± S.E.M. (*n* = 10 per condition). (**C**) Graphic representation of tumour weights mean ± S.E.M. obtained after mice were killed (*n* = 10 per condition). For each condition a picture of a representative tumour is shown. *P < 0.05 shPRUNE2 *versus* pLKO; ***P < 0.001 shKLRC3 *versus* pLKO; shCHI3L1 *versus* pLKO. (**D**) Upper panel: Immunohistochemistry of tumour sections obtained after staining with haematoxylin eosin saffron (HES) (upper part) or Ki67 proliferation marker (lower part). For each condition a picture of a representative tissue section is shown. Bottom panel: Graphic representation of the positive Ki67 staining percentage obtain in the tumour sections for each condition (more than 4 fields per condition). ***P < 0.001 shRNA *versus* pLKO control; $$$*P* < 0.001.

To determine the putative role of *CHI3L1*,* KLRC3* and *PRUNE2* genes in glioblastoma aggressiveness, we have assessed the three shRNA cell lines tumourigenic potential. shRNA and pLKO cells were engrafted subcutaneously in nude mice (*N* = 10 per group). After 28 days the mice were killed and the obtained tumours analysed. In 100% of the cases, the engraftment of the pLKO control cells leads to the formation of a tumour (Fig. 3A). The percentage of tumour formation is reduced when the shRNA cells were engrafted compared to the pLKO control cells. Interestingly, the shKLRC3 cells have formed tumour mass in only 30% of the engraftment meaning that the extinction of the *KLRC3* gene could reduce the tumourigenicity of the U87‐MG cells (Fig. 3A).

pLKO tumours grown faster than shRNA cells grafted tumours as demonstrated by the delay for tumour detection. Indeed, tumours were detected 15 days after implantation of pLKO cells whereas tumours obtained with shRNA inactivated cells were not detected before 24 days after engraftment (Fig. 3B). We then analyse the tumours after mice were killed. Weights of shRNA cells grafted tumours were significantly lower than control ones (*P* < 0.05 for shPRUNE2 tumours and *P* < 0.001 for shCHI3L1 and shKLRC3 tumours) (Fig. 3C). Tumour volumes from shRNA cells grafted were reduced compared to control cells (Fig. 3B). Furthermore, the tumour volumes for shKLRC3 as well for shCHI3L1 grafts (50 mm^3^) were significantly reduced compared to shPRUNE2 grafts (150 mm^3^) suggesting that *KLRC3* and *CHI3L1* might be of prime importance in tumourigenesis process. Finally, to analyse the proliferation status of the tumours, Ki67 staining of paraffin embedded tumours has been done. The clear and significant decrease in Ki67 staining demonstrates that shKLRC3 tumours are composed by less proliferative cells than the three other ones (Fig. 3D). These results confirmed those obtained *in vitro*.

### KLRC3 role in glioblastoma aggressiveness is linked to DAP12/GSK3β signalling pathway activation

**Figure 4:**
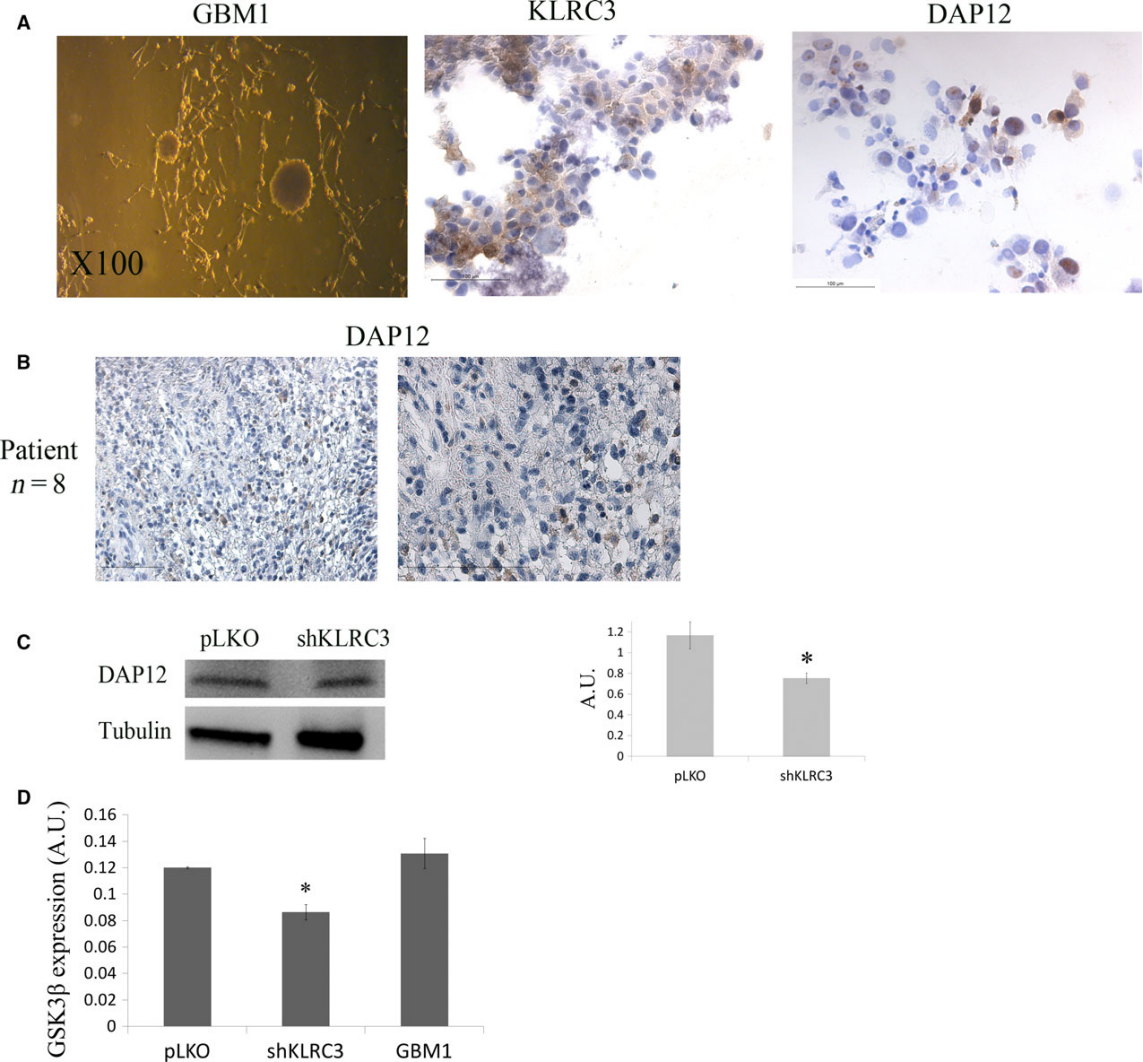
KLRC3 mechanism of action in GBM involves DAP12/GSK3β pathway. (**A**) Staining of KLRC3 and DAP12 proteins in a primary culture derived from a patient tumour. (**B**) DAP12 staining in tissue section from patient's tumours revealing presence of DAP12 in all the GBMs analysed (*n* = 8). (**C**) Left panel: DAP12 protein expression level analysed by western blot in control cells and shKLRC3 cells. Right panel: Graphic representation of the DAP12 western blot quantification, *P < 0.05 (*n* = 3). (**D**) Graphic representation of the protein quantification of GSK3β obtained from the proteome profiler array. *P < 0.05: shKLRC3 *versus* pLKO or GBM1.

Although U87 cell line is the most commonly used cell line in glioma field, we were aware that a cell line is not the best representation of a disease. However, the use of a cell line model is a first step to decipher potential mechanisms involved in the studied disease. To further understand the mechanism involving *KLRC3* in glioblastoma tumourigenesis, we focused on DAP12 protein which has been shown to form a heterodimeric complex with KLRC3 and analysed its expression directly in patients’ samples. We used a primary culture derived from a patient tumour and show that both KLRC3 and DAP12 proteins are expressed in the primary culture (Fig. 4A). We then studied the DAP12 expression directly in tumour samples from patients and found it strongly expressed in all the tumour samples analysed (*n* = 8) confirming the presence of DAP12 in GBMs cells (Fig. 4B). Moreover, DAP12 protein expression is founded reduced in shKLRC3 cells compared to control ones by western blot analysis (Fig. 4C) validating a role of the KLRC3‐DAP12 complex in GBM aggressiveness. Since DAP12 is able to activate different signalling pathways, we used a proteome profiler array to compare the expression level of signalling proteins involved in cell proliferation between the control cell line, the shKLRC3 cells and the primary culture derived from a patient tumour (GBM1) (Fig. S2). Among the signalling proteins analysed, we choose to focus on the ones involved in cell radioresistance since we demonstrated that KLRC3 is involved in this glioblastoma phenotype. The obtained results show a significant reduction in the GSK3β protein in shKLRC3 compared to the control cell line and the primary culture derived from a patient's tumour (Fig. 4D).

## Discussion

We have previously described that different glycosylation‐related genes are overexpressed in undifferentiated glioblastoma cells leading to a specific glyco‐signature of CSC and suggesting a role of these genes in ‘stem phenotype’ and cancer hallmark 4. According to the role of CSC in glioblastomas aggressiveness and recurrence, we focused on the respective involvement of these genes in glioblastomas aggressiveness and tumourigenicity. Among these genes, three specific ones: *CHI3L1*,* KLRC3* and *PRUNE2* were studied. Their silencing by shRNA in U87‐MG cell lines point out that only *KLRC3* gene affects specifically proliferation, radiosensitivity, invasion ability, self‐renewal properties and tumourigenicity *in vivo*.

CHI3L1 also named YKL‐40 is strongly expressed in high grade gliomas 8. CHI3L1 was previously reported in tumour angiogenesis 6, 9, 10, in glioma invasion process 11 and radioresistance 10. We have previously reported that CHI3L1 was overexpressed in undifferentiated glioblastoma stem cells (GSC) from U87‐MG and U251 cell lines and in gliomaspheres from primary cell culture derived from patients 4. Although *CHI3L1* plays a role in cell proliferation, its inactivation does not affect the cell proliferation after radiation, meaning that its inactivation does not sensitizes the cells to treatment. In contrast, *KLRC3* silencing decreases cell proliferation significantly and even more significantly after cell radiation, suggesting its involvement in glioma radiosensitivity. The function of *KLRC3* is not restricted to its effect on radiosensitivity but also concern glioblastoma self‐renewal property which is significantly decreased compared to *CHI3L1* extinction. These two roles are closely linked to the tumour aggressiveness and are confirmed with the reduce tumourigenicity observed after shKLRC3 cell grafts *in vivo*.

Concerning *PRUNE2*, this gene is up‐regulated in prostate cancer where it plays a role in post‐endocytic trafficking 5 and in metastasis 12. This gene, known to be highly expressed in mature nerve tissue 13 has never been described in gliomas except in our previous report 4. Radiation did not significantly modify proliferation of shPRUNE2 cells while shKLRC3 cell proliferation was decreased, suggesting KLRC3 involvement in glioblastoma cells radioresistance mechanism. The increase in radiation resistance might be because of the selection of CSCs 14. Accordingly, *KLRC3* silencing induces a significant decrease in glioblastoma cells self‐renewal ability suggesting that *KLRC3* is of prime importance in the maintenance of CSC subpopulation. This major function is confirmed by the predominant decrease in colony‐forming cells following its gene silencing compared to those of *PRUNE2* and *CHI3L1*. These datas concerning *KLRC3* involvement in glioblastoma aggressiveness are supported by the decrease in tumour growth after shKLRC3 cells engraftment *in vivo* compared to control cells. By using public databases, we founded that KLRC3 gene expression level increased more than 1.7 times in glioma samples compare to normal tissue (from GENT database: Gene Expression across Normal and Tumour tissue) 15. Moreover, in a cohort of 154 patients with glioblastoma, KLRC3 has been founded mutated in 6% of the cases. By comparing the median survival of the patients included in the cohort, we founded a strong increase from 13.3 months for the patients without mutation to 25.4 months of survival for the patients having a mutated KLRC3 (from the TCGA analysis: The Cancer Genome Atlas) 16.

KLRC3 (also named NKG2E), is related to the NKG2x family, and has been described as forming a heterodimeric complex with CD94 17, 18. This complex interacts with HLA‐E 17, MHC class 1b protein, known to be involved in immunosuppressive mechanisms, which is highly expressed in several tumours such as melanoma 19, colon cancer 20 and glioblastoma 21. Recent findings demonstrate that HLA‐E is overexpressed in several GSCs culture and contributes to inhibit NK cells and promotes immune escape 22.

This heterodimeric complex can also recruit DAP12, an adaptor protein 18. DAP12 expression associated with KLRC3 has been previously reported in breast cancer and is correlated with a higher risk of metastasis and invasive process in bone and liver 23. The expression of DAP12 was also shown in T98G glioblastoma cells 24. However, KLRC3 function in glioblastoma is still unknown. The intracellular localization of KLRC3 18 suggests that this immune receptor might interact with other signalling pathways. Since KLRC3 has never been linked to glioma, its mechanism of action in this model remains unknown. We show in this study the strong expression of DAP12 in primary culture derived from tumour patient and in tumour tissues. DAP12 protein can lead to the activation of different signalling pathways which one of them is the activation of GSK3β protein 25. The decrease in DAP12 expression level in shKLRC3 cells associated with the significant decrease in the GSK3β protein strongly supports the hypothesis of a link between KLRC3 and DAP12 in the regulation of the glioblastoma aggressiveness, particularly in the radioresistance phenotype. GSK3β has been linked to glioma invasion ability 26 and its inhibition leads to an increase in glioma sensitivity to Temozolomide 27 and to radiotherapy 28. It has been also demonstrated that inhibition of GSK3β in GSC isolated from GBM patient samples attenuates cell proliferation, suggesting GSK3β as a potential therapeutical target in GSC 29.

These results point out a new role and mechanism of action of *KLRC3* in glioblastoma cells and reinforce our previous results evidencing *KLRC3* overexpression in glioblastoma CSCs. Taken together these findings suggest that *KLRC3* plays a major role in glioblastoma aggressiveness.

## Conflict of interest

The authors declare that they have no conflicts of interest with the contents of this article.

## Supporting information


**Figure S1** Representative western blot validation of the different clones used for each shRNA before selection of the most efficient one (based on the reduction in the protein expression level) for each analysed gene.

**Figure S2** Graphic representation of the quantification of the results obtained with the Proteome profiler array on pLKO, shKLRC3 and GBM1 (primary culture).
