# Supplementary figures and images for: KLRC3, a Natural Killer receptor gene, is a key factor involved in glioblastoma tumourigenesis and aggressiveness
Source: J Cell Mol Med. 2016 Sep 19;21(2):244–53. doi: 10.1111/jcmm.12960 (PMC5264145; doi:10.1111/jcmm.12960)

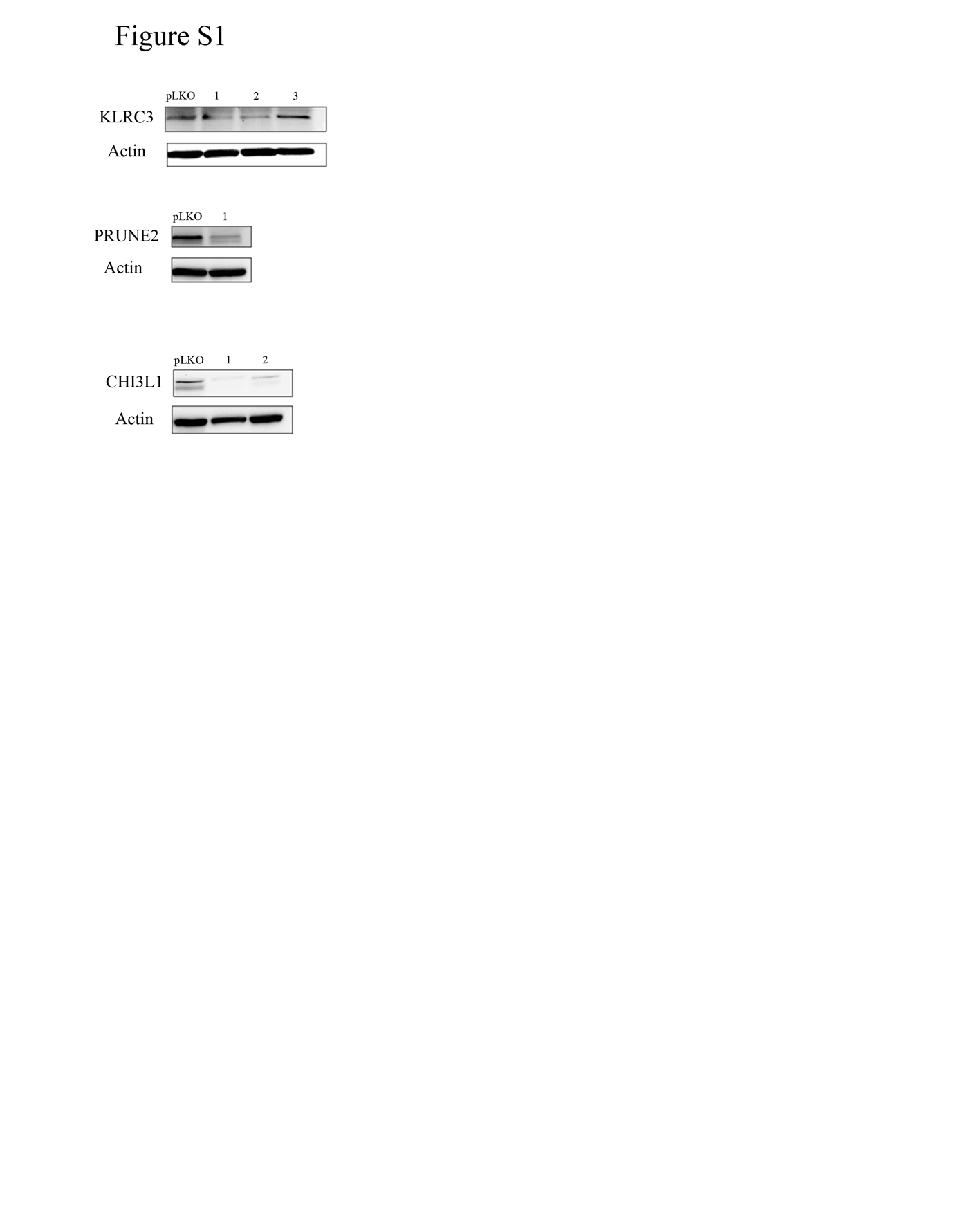

Supplement: Supplementary file 1 — Figure S1 Representative western blot validation of the different clones used for each shRNA before selection of the most efficient one (based on the reduction in the protein expression level) for each analysed gene. [file JCMM-21-244-s001.tif]

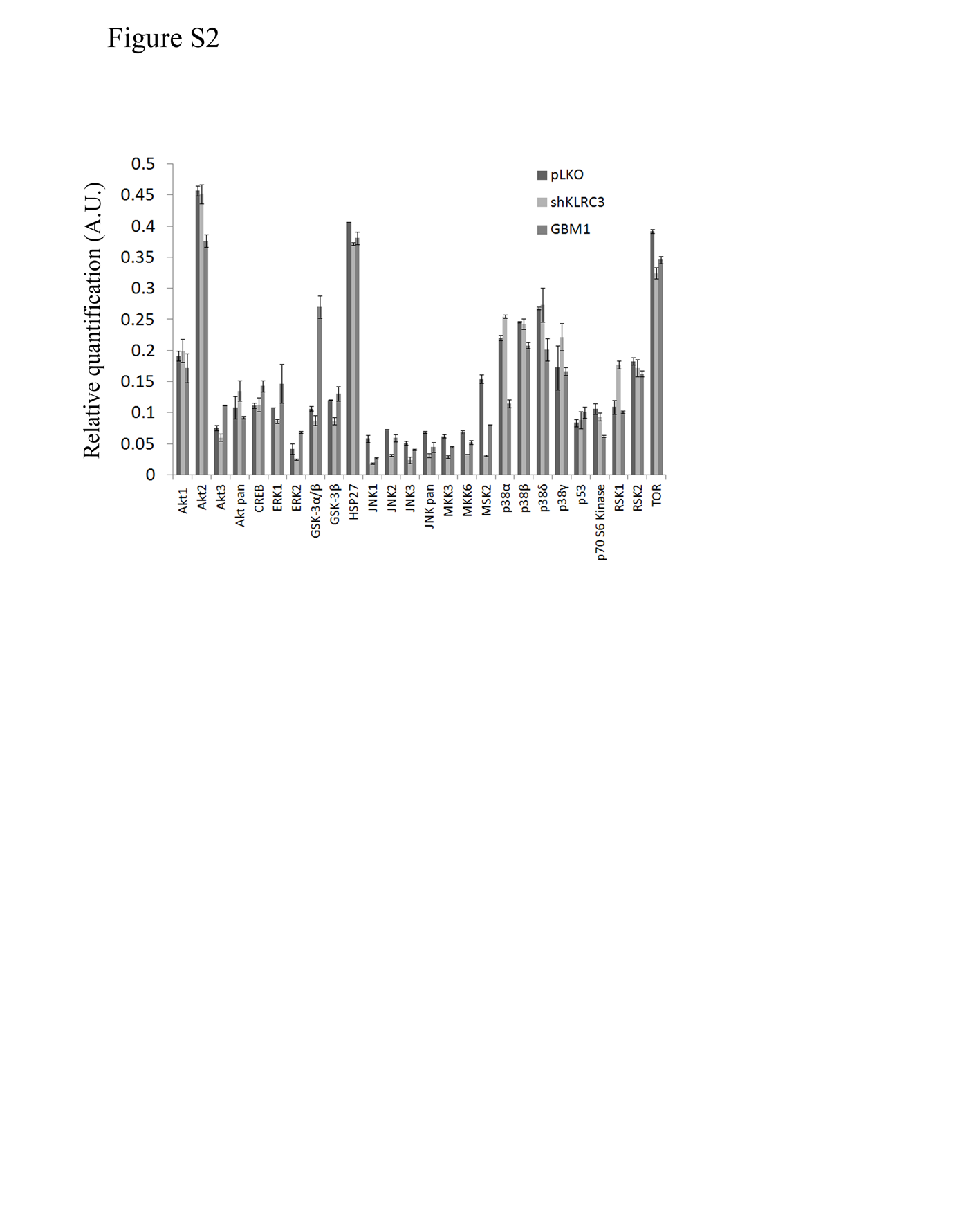

Supplement: Supplementary file 2 — Figure S2 Graphic representation of the quantification of the results obtained with the Proteome profiler array on pLKO, shKLRC3 and GBM1 (primary culture). [file JCMM-21-244-s002.tif]
